# Hemorrhagic Pericarditis as a Harbinger of Giant Cell Arteritis Relapse

**DOI:** 10.1016/j.jaccas.2025.104627

**Published:** 2025-08-06

**Authors:** Rohanti Ravikulan, Rupini Raja, Jasmine Chan, Muditha Jeyaweera, Vijay Turaka, Mihir D. Wechalekar, Joanne Eng-Frost

**Affiliations:** a Department of Cardiology, Flinders Medical Centre, Adelaide, Australia; b Rheumatology Unit, Flinders Medical Centre, Flinders University, Adelaide, Australia; c Department of General Medicine, Flinders Medical Centre, Adelaide, Australia; d College of Medicine and Public Health, Flinders University, Adelaide, Australia

**Keywords:** effusion, giant cell arteritis, hemorrhagic, pericardial disease, pericardial effusion, pericarditis, vasculitis

## Abstract

**Background:**

Pericarditis in patients with giant cell arteritis (GCA) has been described in literature but is infrequently recognised as an initial manifestation, posing diagnostic challenges.

**Case summary:**

A 71-year-old male presented with pericarditis as the sole clinical manifestation on a background of previously treated GCA. Despite the absence of hallmark symptoms, he was confirmed to have medium-vessel vasculitis on a fluorodeoxyglucose-positron emission tomography scan.

**Discussion:**

This case highlights the importance of considering pericarditis and pericardial effusions as potential manifestations of GCA even in the absence of typical symptoms to ensure timely management and minimize complications and underscores the need for further data on treatment specific to GCA-related pericarditis.

**Take-Home Messages:**

Pericarditis may be an atypical presenting symptom of GCA, occurring in the absence of typical symptoms. At present, there are limited data on the treatment of GCA-related pericarditis despite its recognition as a manifestation of the disease.

## Graphical Abstract

**Figure:**
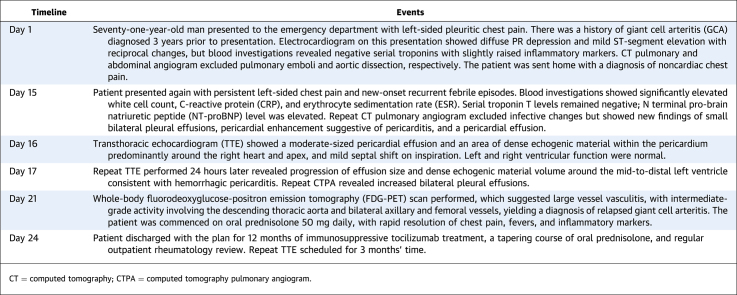

## History of Presentation

A 71-year-old man presented to the emergency department with a few hours of worsening left-sided pleuritic chest pain radiating to the neck, jaw, and the upper abdomen. Twelve-lead electrocardiogram on admission showed diffuse PR depression and mild ST-segment elevation with reciprocal changes in lead aVR. The initial workup revealed anemia, mildly elevated inflammatory markers (white cell count [WCC], 14.86 × 10^9^/L [normal range: 4-11 × 10^9^/L], absolute neutrophil count [ANC], 11.74 × 10^9^/L [normal range: 1.8-7.5 × 10^9^/L], and C-reactive protein [CRP], 29.7 mg/L [normal range: 0-8.0 mg/L]), and negative serial troponin T levels (9 ng/L and 10 ng/L [normal range: <16 ng/L]). Computed tomography (CT) pulmonary and abdominal angiogram excluded pulmonary emboli and aortic dissection, respectively, however were suggestive of omental inflammation and/or infarct. A presumptive diagnosis of noncardiac chest pain was made, and the patient was discharged home with simple analgesia.

**Take-Home Messages**

• Pericarditis may be an atypical presenting symptom of giant cell arteritis, occurring in the absence of typical symptoms.
• At present, there are limited data on the treatment of giant cell arteritis–related pericarditis despite its recognition as a manifestation of the disease.

He subsequently re-presented to the emergency department 2 weeks later with persistent left-sided chest pain and new-onset recurrent febrile episodes. Investigations revealed significantly elevated inflammatory markers (WCC 14 × 10^9^/L, ANC, 11.10 × 10^9^/L, CRP 338.7 mg/L, erythrocyte sedimentation rate 115 mm [normal range: 1-15 mm]). Serial troponin T levels remained negative (14 ng/L), although the N terminal pro-brain natriuretic peptide level was elevated (1,085 ng/L [normal range: 0-124 ng/L]). Repeat CT pulmonary angiogram excluded infective changes, and however, revealed new findings of small bilateral pleural effusions, pericardial enhancement suggestive of pericarditis, and a pericardial effusion.

## Past Medical History

The patient had a history of giant cell arteritis (GCA) diagnosed 3 years prior to presentation. His presenting complaints at the time were scalp and neck pain, and pain in the jaw on opening the mouth (without claudication). Temporal artery biopsy (TAB) was negative for vasculitis; however, subsequent magnetic resonance imaging (GCA protocol) findings were suggestive of large vessel vasculitis. He was managed with a 9-month course of oral prednisolone; subcutaneous tocilizumab administered weekly for 20 months, which was completed 15 months prior to current presentation; and regular outpatient rheumatology reviews. He remained in remission with no disease relapses. Other medical history included hypertension managed with telmisartan and amlodipine, type 2 diabetes mellitus managed with oral metformin, left metatarsal joint gout managed with regular allopurinol, and colchicine during gout flares.

## Investigations and Management

**Figure 1:**
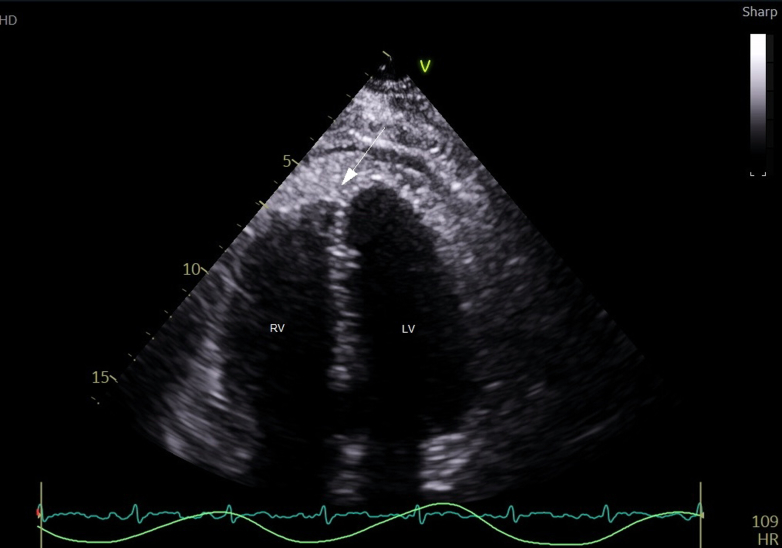
Initial Transthoracic Echocardiogram Transthoracic echocardiogram (TTE) apical 4-chamber view, performed on day 16 of admission, showing dense echogenic material in the pericardium (white arrow), indicative of a pericardial effusion, adjacent to the right ventricle. LV = left ventricle; RV = right ventricle.

**Figure 2:**
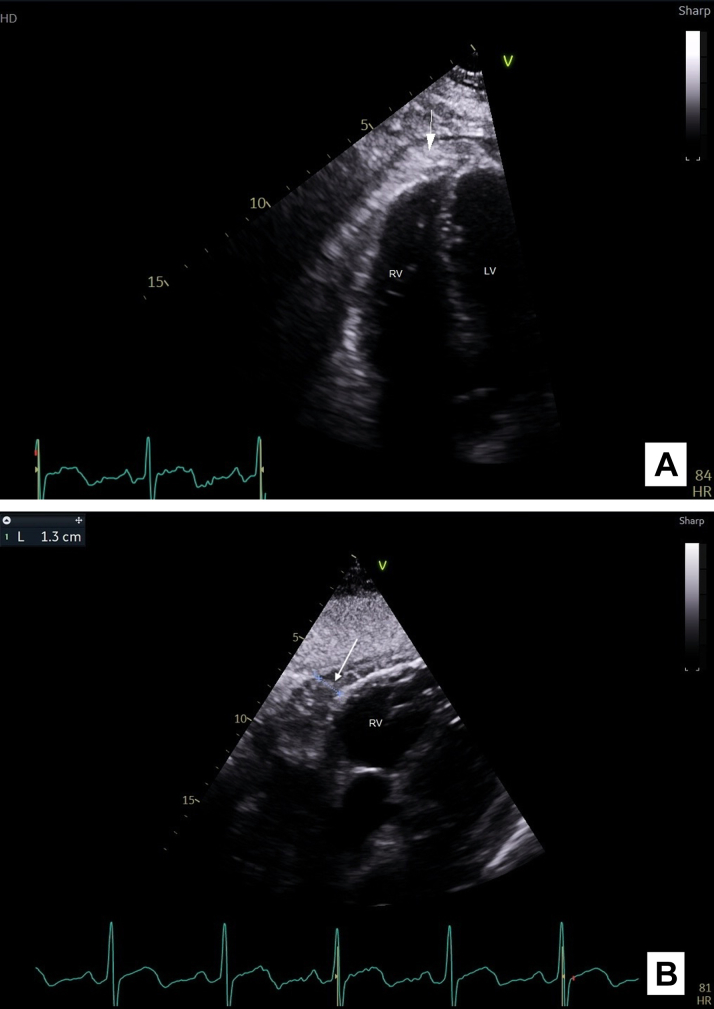
Repeat Transthoracic Echocardiogram Transthoracic echocardiogram (TTE) performed 24 hours following the initial TTE (day 17 of admission), apical 4-chamber view (A) and subcostal view (B) showing dense echogenic material in the pericardium (white arrows) adjacent to the right ventricle, increased in size compared to previous image. LV = left ventricle; RV = right ventricle; TTE = transthoracic echocardiogram.

Initial transthoracic echocardiogram (TTE) revealed a moderate-sized pericardial effusion measuring up to 1.8 cm adjacent to the left ventricle, 1.1 cm apically, and 1.3 adjacent to the right heart. Furthermore, there was an area of dense echogenic material within the pericardium predominantly around the right heart and apex and mild septal shift on inspiration (Figure 1). Left and right ventricular functions were normal. Repeat TTE performed 24 hours later revealed progression of effusion size and dense echogenic material volume around the mid-to-distal left ventricle consistent with hemorrhagic pericarditis (Figure 2). This progression was likely exacerbated by the initiation of subcutaneous enoxaparin 40 mg daily for venous thromboembolism prophylaxis on admission. Enoxaparin was discontinued, and the patient was commenced on colchicine 500 μg twice daily. Repeat TTE 24 hours later showed no significant change in the size of the pericardial effusion.

During his admission, the patient developed symptomatic atrial fibrillation with a rapid ventricular response, for which he was commenced on metoprolol 25 mg twice daily. Anticoagulation was deferred despite a CHA_2_DS_2_-VASc score 5, due to the suspicion of hemorrhagic pericarditis with a moderate-sized effusion. There was further clinical deterioration over the next 24 hours, with worsening dyspnea and recurrent febrile episodes. Repeat CT pulmonary angiogram revealed increased bilateral pleural effusions. Comprehensive infective screening was performed, including HIV serology, *Legionella* serology and urinary antigen, Rickettsia serology, Q fever serology and polymerase chain reaction, hepatitis B and C serology, QuantiFERON gold, Strongyloides serology, syphilis serology and stool enteric polymerase chain reaction, and abdominal and pelvic CT. These investigations were all negative.

**Figure 3:**
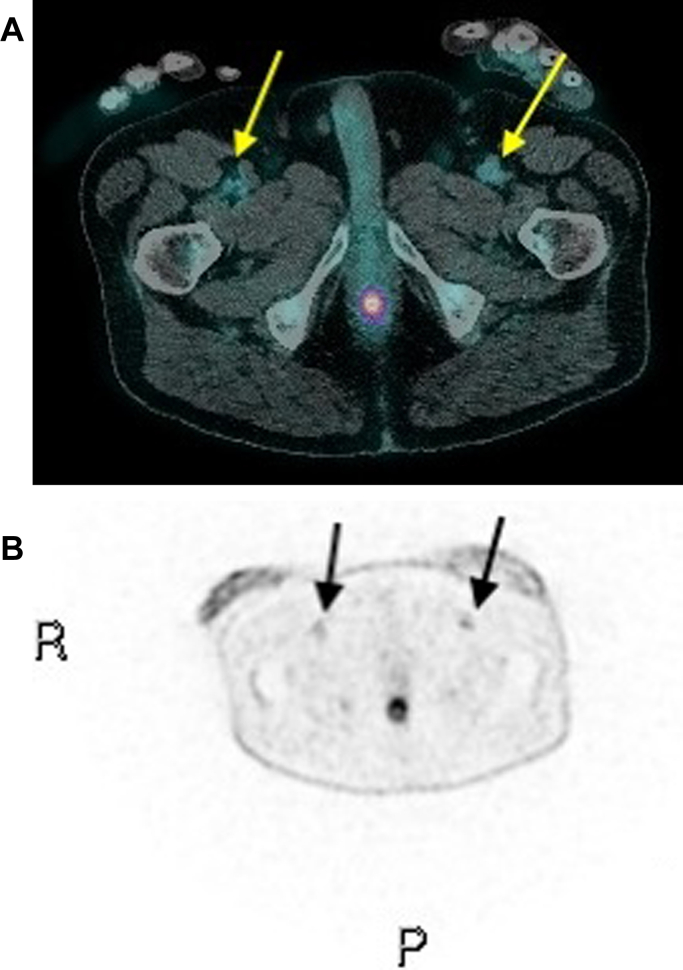
Fluorodeoxyglucose-Positron Emission Tomography Scan Whole-body fluorodeoxyglucose-positron emission tomography (FDG-PET) scan performed on day 21; fused (A) and native (B) images showing enhancement of bilateral femoral arteries (yellow arrows, black arrows), indicative of medium-vessel vasculitis. P = posterior; R = right.

Cardiac magnetic resonance imaging was performed, which confirmed a large, hemorrhagic pericardial effusion. In consultation with the infectious diseases and rheumatology teams, the patient underwent whole-body fluorodeoxyglucose-positron emission tomography (FDG-PET) scan. FDG-PET was highly suggestive of large vessel vasculitis, with intermediate-grade activity involving the descending thoracic aorta and bilateral axillary and femoral vessels (Figure 3). The patient was commenced on oral prednisolone 50 mg daily, with rapid resolution of chest pain, fevers, and inflammatory markers within 72 hours of commencement (WCC 10.93 × 10^9^/L, ANC 7.6 × 10^9^/L, CRP 17.5 mg/L). He was discharged 3 days later, with the plan for a further 12 months of immunosuppressive tocilizumab treatment, a tapering course of oral prednisolone, and regular outpatient rheumatology review. A repeat TTE is planned in 3 months from the time of discharge to monitor the progress of the hemorrhagic pericardial effusion.

## Discussion

GCA is a systemic autoimmune vasculitis primarily affecting large- and medium-sized arteries. It typically occurs in patients over the age of 50 years. Typical symptoms of a GCA flare include headache, scalp tenderness, jaw claudication, and vision loss, alongside constitutional symptoms including fever and malaise.^1^ Cardiac complications associated with vasculitis including pericarditis, myocarditis, aortic aneurysm or dissection, and coronary vasculitis are less common but can occur in up to 5% of patients with GCA.^2^ Studies have identified an independent association between GCA and the occurrence of pericarditis^1^ and pericardial effusion.^3^ Furthermore, a systematic review examining concomitant pericardial disease with GCA, and polymyalgia rheumatica (PMR; a condition often associated with GCA), revealed that pericardial disease was the presenting feature in 37% of such patients.^4^ Furthermore, in a retrospective cohort study of 250 patients with GCA, 23 (9.2%) patients presented with pericardial effusion at diagnosis, with 6 cases being compatible with pericarditis (including one myopericarditis), associated with raised inflammatory markers.^5^ These studies suggest that pericardial involvement in GCA, although atypical, is not unrecognized.

Pulmonary manifestations of GCA are less common than cardiac complications and include pulmonary nodules, pulmonary artery thrombosis, pulmonary infarction, pleural effusion, alveolar hemorrhage, and lymphocytic alveolitis.^6^ Pleural effusions are typically unilateral and exudative^6^ and may be the presenting symptom of GCA.^3^^,^^6^ While there have been documented cases of simultaneous pericarditis and pleural effusions, as described in our report, this is the first documented case of hemopericardium as the predominant presenting feature in a patient with GCA.

Given the highly variable nature of presenting symptoms, timely and accurate diagnosis of GCA can be challenging. TAB remains the gold standard for establishing diagnosis. However, its accuracy is highly dependent on disease distribution and skip lesions, resulting in false negative rates of up to 40%, particularly in cases predominantly affecting large vessels.^7^ CT angiography is commonly used to diagnose large-vessel vasculitis. However, its utility is limited during the inflammatory phase, with a sensitivity of only 73% for both CT and magnetic resonance angiography.^7^ FDG-PET imaging offers a higher diagnostic yield, with a sensitivity of 83.5% in patients with negative TAB and 100% in patients with extracranial large-vessel involvement.^8^ In our case, FDG-PET imaging was critical in identifying active inflammation across multiple large-vessel beds, thus establishing a diagnosis. However, caution is needed when using FDG-PET to monitor GCA disease activity as there have been reports indicating persistent radiotracer uptake despite clinical remission.^7^ In their 2021 guideline, the American College of Rheumatology recommends long-term clinical monitoring of patients with GCA, which may comprise history, examination, laboratory tests, and imaging but does not make specific recommendations on the frequency or method of monitoring.^9^ Based on current literature, ultrasound may be a useful tool in monitoring disease activity following initial treatment.^10^

There is a paucity of literature to guide the management of GCA-related pericarditis, which is likely to be interleukin-6-driven and may not respond to treatment strategies typically used for autoimmune-related pericarditis that occurs as a result of rheumatoid arthritis or systemic lupus erythematosus, due to differing underlying pathologic processes. interleukin-6 inhibitors, particularly tocilizumab, in conjunction with glucocorticoids remains the current mainstay of pharmacotherapy in GCA.^1^^,^^3^ In some cases, glucocorticoid therapy alone was effective in preventing recurrence of GCA-related pericardial effusion.^5^

Other treatment options are limited. Colchicine is commonly used in the management of acute pericarditis and recurrent pericarditis; however, the efficacy of colchicine in GCA-related pericarditis has not yet been evaluated.^1^ Although tocilizumab is of proven efficacy in GCA, its efficacy in GCA-related pericarditis awaits formal evaluation. Some small-scale studies have also investigated the use of interleukin-1 inhibitors including canakinumab and anakinra in the context of idiopathic recurrent pericarditis; however, evidence supporting their use in GCA-related pericarditis remains limited.^1^

## Conclusions

Our case highlights the complexities and challenges associated with diagnosing and managing the multifaceted disease of GCA. At present, there are no comprehensive guidelines outlining effective treatment strategies. Management is further complicated by an incomplete understanding of the pathophysiological mechanisms underlying GCA-related pericarditis. Our case report underscores the need for vigilance, prospective detection, and investigation of the efficacy of GCA-treatment protocols and monitoring strategies for this rare but significant complication.

## Funding Support and Author Disclosures

The authors have reported that they have no relationships relevant to the contents of this paper to disclose.

**Visual Summary:** Timeline of the Case

Timeline	Events
Day 1	Seventy-one-year-old man presented to the emergency department with left-sided pleuritic chest pain. There was a history of giant cell arteritis (GCA) diagnosed 3 years prior to presentation. Electrocardiogram on this presentation showed diffuse PR depression and mild ST-segment elevation with reciprocal changes, but blood investigations revealed negative serial troponins with slightly raised inflammatory markers. CT pulmonary and abdominal angiogram excluded pulmonary emboli and aortic dissection, respectively. The patient was sent home with a diagnosis of noncardiac chest pain.
Day 15	Patient presented again with persistent left-sided chest pain and new-onset recurrent febrile episodes. Blood investigations showed significantly elevated white cell count, C-reactive protein (CRP), and erythrocyte sedimentation rate (ESR). Serial troponin T levels remained negative; N terminal pro-brain natriuretic peptide (NT-proBNP) level was elevated. Repeat CT pulmonary angiogram excluded infective changes but showed new findings of small bilateral pleural effusions, pericardial enhancement suggestive of pericarditis, and a pericardial effusion.
Day 16	Transthoracic echocardiogram (TTE) showed a moderate-sized pericardial effusion and an area of dense echogenic material within the pericardium predominantly around the right heart and apex, and mild septal shift on inspiration. Left and right ventricular function were normal.
Day 17	Repeat TTE performed 24 hours later revealed progression of effusion size and dense echogenic material volume around the mid-to-distal left ventricle consistent with hemorrhagic pericarditis. Repeat CTPA revealed increased bilateral pleural effusions.
Day 21	Whole-body fluorodeoxyglucose-positron emission tomography (FDG-PET) scan performed, which suggested large vessel vasculitis, with intermediate-grade activity involving the descending thoracic aorta and bilateral axillary and femoral vessels, yielding a diagnosis of relapsed giant cell arteritis. The patient was commenced on oral prednisolone 50 mg daily, with rapid resolution of chest pain, fevers, and inflammatory markers.
Day 24	Patient discharged with the plan for 12 months of immunosuppressive tocilizumab treatment, a tapering course of oral prednisolone, and regular outpatient rheumatology review. Repeat TTE scheduled for 3 months’ time.

CT = computed tomography; CTPA = computed tomography pulmonary angiogram.
